# Supplementary material for: Analysis of PPARγ Signaling Activity in Psoriasis
Source: Int J Mol Sci. 2021 Aug 10;22(16):8603. doi: 10.3390/ijms22168603 (PMC8395241; doi:10.3390/ijms22168603)
Supplement: Supplementary file 1 [file ijms-22-08603-s001.zip › Supplemental materials_Analysis of PPARg signaling activity in psoriasis/Pathway models/Models images and html files/Differentiation of psoriatic T cells/30079.html]

TBX21


# Protein TBX21

|  |  |
| --- | --- |
| URN | urn:agi-llid:29939 |
| Total Entities | 0 |
| Connectivity | 2180 |
| Name | TBX21 |
| Description | T-box 21 |
| Notes | This gene is a member of a phylogenetically conserved family of genes that share a common DNA-binding domain, the T-box. T-box genes encode transcription factors involved in the regulation of developmental processes. This gene is the human ortholog of mouse Tbx21/Tbet gene. Studies in mouse show that Tbx21 protein is a Th1 cell-specific transcription factor that controls the expression of the hallmark Th1 cytokine, interferon-gamma (IFNG). Expression of the human ortholog also correlates with IFNG expression in Th1 and natural killer cells, suggesting a role for this gene in initiating Th1 lineage development from naive Th precursor cells. [provided by RefSeq, Jul 2008] |
| Primary Cell Localization | Nucleus |
| Class | Transcription factor |
| ObjectType | Protein |

---

|  |  |
| --- | --- |
| Pathway | CD8+ T-Cell Activation |
|  | Th1-Cell Differentiation |
|  | AHR Signaling in Tr1 Cells Function |
|  | Proteins Involved in Atherosclerosis |
|  | Th1-Cells Activation and Proliferation in Atherosclerosis |
|  | Lymphocyte Mediated Myocardial Injury in Myocarditis |
|  | Proteins Involved in Myocarditis |
|  | Atopic Dermatitis |
|  | T-Cells Differentiation Block in Psoriasis |
|  | Th1-Cell Function in Systemic Lupus Erythematosus |
|  | Vitiligo |
|  | T-Cell Cytotoxic Response against Melanocytes in Vitiligo |
|  | Proteins Involved in Myasthenia Gravis |
|  | CD4+T-Cell Response in Celiac Disease |
|  | Th1-Cell Activation in Crohn's Disease |
|  | Proteins Involved in Helicobacter Infections |
|  | Proteins Involved in Ulcerative Colitis |
|  | Proteins Involved in Systemic Scleroderma |
|  | Proteins Involved in Spontaneous Abortion |
|  | Proteins Involved in Glomerulonephritis |
|  | CD80 -> ATF/CREB/CREBBP Expression Targets |
|  | IFNA1/IFNR Expression Targets |
|  | IFNG/IFNR Expression Targets |
|  | IL27R -> STAT Signaling |
|  | IL21 Expression Targets |
|  | IL15 Expression Targets |
|  | NOTCH -> Expression Targets in Thymus |
|  | PRL/PRLR Expression Targets |
|  | PRL/GHR -> STAT Expression Targets |
|  | IL1B Expression Targets |
|  | IL1A Expression Targets |
|  | PPAR Psoriasis |
|  | prarg negative regulators, ps-positive |
|  | pprarg neg, uknown targets, ps-positive |
|  | prarg neg,ukn expres targets, ps-positive |
|  | PPARG negative regulators |
|  | PPARG negative targets |
|  | pparg expr target\_regulators |
|  | Neighbors of disease exacerbation |
|  | Neighbors of tumor progression |
|  | Neighbors of cancer progression |
|  | Neighbors of overall survival |
|  | Neighbors of disease severity |
|  | Neighbors of tumor size |
|  | Neighbors of lesion size |
|  | Neighbors of mortality |
|  | Neighbors of insulin sensitivity |
|  | Neighbors of bacterial load |
|  | Neighbors of therapeutic efficacy |
|  | Neighbors of lethality |
|  | Neighbors of disease-free survival |
|  | Neighbors of tumor response |
|  | Neighbors of macrophage count |
|  | Neighbors of long-term survival |
|  | Neighbors of body weight |
|  | Neighbors of morbidity |
|  | Neighbors of exhaustion |
|  | Neighbors of early mortality |
|  | Neighbors of viral load |
|  | Neighbors of blood pressure |
|  | Neighbors of median survival |
|  | Neighbors of fasting plasma glucose |
|  | Neighbors of antimetastatic activity |
|  | Neighbors of gastric cancer risk |
|  | Neighbors of disease exacerbation |
|  | Neighbors of tumor progression |
|  | Neighbors of cancer progression |
|  | Neighbors of overall survival |
|  | Neighbors of disease severity |
|  | Neighbors of tumor size |
|  | Neighbors of lesion size |
|  | Neighbors of mortality |
|  | Neighbors of insulin sensitivity |
|  | Neighbors of bacterial load |
|  | Neighbors of therapeutic efficacy |
|  | Neighbors of lethality |
|  | Neighbors of disease-free survival |
|  | Neighbors of tumor response |
|  | Neighbors of macrophage count |
|  | Neighbors of long-term survival |
|  | Neighbors of body weight |
|  | Neighbors of morbidity |
|  | Neighbors of exhaustion |
|  | Neighbors of early mortality |
|  | Neighbors of viral load |
|  | Neighbors of blood pressure |
|  | Neighbors of median survival |
|  | Neighbors of fasting plasma glucose |
|  | Neighbors of antimetastatic activity |
|  | Neighbors of gastric cancer risk |
|  | Neighbors of cell development |
|  | Neighbors of immune response |
|  | Neighbors of cell population |
|  | Neighbors of T-cell development |
|  | Neighbors of inflammatory response |
|  | Neighbors of innate immune response |
|  | Neighbors of cell function |
|  | Neighbors of cell formation |
|  | Neighbors of adaptive immune response |
|  | Neighbors of epithelial to mesenchymal transition |
|  | Neighbors of immunity |
|  | Neighbors of cell survival |
|  | Neighbors of cell phenotype |
|  | Neighbors of T-cell proliferation |
|  | Neighbors of cell adhesion |
|  | Neighbors of T-cell activation |
|  | Neighbors of tumor growth |
|  | Neighbors of cell count |
|  | Neighbors of cell infiltration |
|  | Neighbors of cell interaction |
|  | Neighbors of adipogenesis |
|  | Neighbors of pregnancy |
|  | Neighbors of neutrophil recruitment |
|  | Neighbors of cell migration |
|  | Neighbors of leukocyte migration |
|  | Neighbors of cell invasion |
|  | Neighbors of heart function |
|  | Neighbors of hemopoiesis |
|  | Neighbors of T-cell function |
|  | Neighbors of immune system function |
|  | Neighbors of transcription activation |
|  | Neighbors of senescence |
|  | Neighbors of T-cell response |
|  | Neighbors of tumor immunity |
|  | Neighbors of macrophage function |
|  | Neighbors of cellular immune response |
|  | Neighbors of cellular senescence |
|  | Neighbors of macrophage differentiation |
|  | Neighbors of cell differentiation |
|  | Neighbors of cell homeostasis |
|  | Neighbors of psoriasis |
|  | Neighbors of inflammatory disease |
|  | Neighbors of rheumatoid arthritis |
|  | Neighbors of atherosclerosis |
|  | Neighbors of fibrosis |
|  | Neighbors of colitis |
|  | Neighbors of arthritis |
|  | Neighbors of inflammatory bowel disease |
|  | Neighbors of metastasis |
|  | Neighbors of carcinogenesis |
|  | Neighbors of multiple sclerosis |
|  | Neighbors of chronic inflammation |
|  | Neighbors of inflammation |
|  | Neighbors of obesity |
|  | Neighbors of vascular disease |
|  | Neighbors of diabetes mellitus |
|  | Neighbors of asthma |
|  | Neighbors of hyperplasia |
|  | Neighbors of chronic obstructive pulmonary disease |
|  | Neighbors of reperfusion injury |
|  | Neighbors of airway inflammation |
|  | Neighbors of hepatitis |
|  | Neighbors of insulin resistance |
|  | Neighbors of infection |
|  | Neighbors of vascular injury |
|  | Neighbors of renal injury |
|  | Neighbors of macrophage infiltration |
|  | Neighbors of neuroinflammation |
|  | Neighbors of death |
|  | Neighbors of type 1 diabetes |
|  | Neighbors of encephalomyelitis |
|  | Neighbors of autoimmune disease |
|  | Neighbors of dermatitis |
|  | Neighbors of heart disease |
|  | Neighbors of lung injury |
|  | Neighbors of immunopathology |
|  | Neighbors of systemic sclerosis |
|  | Neighbors of autoimmunity |
|  | Neighbors of gastric cancer |
|  | Neighbors of experimental autoimmune encephalomyelitis |
|  | Neighbors of colorectal cancer |
|  | Neighbors of cardiac remodeling |
|  | Neighbors of systemic lupus erythematosus |
|  | Neighbors of melanoma |
|  | Neighbors of interstitial fibrosis |
|  | Neighbors of kidney disease |
|  | Neighbors of heart failure |
|  | Neighbors of pulmonary fibrosis |
|  | Neighbors of cancer |
|  | Neighbors of neoplasm |
|  | Neighbors of vasculitis |
|  | Neighbors of diet-induced obesity |
|  | Neighbors of respiratory hypersensitivity |
|  | Neighbors of prostate cancer |
|  | Neighbors of hypertrophy |
|  | Neighbors of hypertension |
|  | Neighbors of allograft rejection |
|  | Neighbors of proteinuria |
|  | Neighbors of epithelial cell |
|  | Neighbors of PBMC |
|  | Neighbors of B-cell |
|  | Neighbors of T-cell |
|  | Neighbors of spleen cell |
|  | Neighbors of lymphoid cell |
|  | Neighbors of blood cell |
|  | Neighbors of colon |
|  | Neighbors of airway |
|  | Neighbors of heart |
|  | Neighbors of gastrointestinal tract |
|  | Neighbors of thymus gland |
|  | Neighbors of reproductive system |
|  | Neighbors of NF-kB family |
|  | Neighbors of JNK |
|  | Neighbors of PPAR |
|  | Neighbors of NFKBI |
|  | Neighbors of PI3K |
|  | Neighbors of STAT family |
|  | Neighbors of mitogen-activated protein kinase |
|  | Neighbors of IFNAR ligand |
|  | Neighbors of transforming growth factor |
|  | Neighbors of TLR |
|  | Neighbors of cytokine |
|  | Neighbors of interferon |
|  | Neighbors of JAK |
|  | Neighbors of ERK1/2 |
|  | Neighbors of ovalbumin |
|  | Neighbors of NF-AT family |
|  | Neighbors of SMAD subfamily |
|  | Neighbors of LXR |
|  | Neighbors of inflammatory cytokine |
|  | Neighbors of IL23 |
|  | Neighbors of T-cell receptor |
|  | Neighbors of IL12 |
|  | Neighbors of Notch |
|  | Neighbors of fibrinogen |
|  | Neighbors of interleukin-35 |
|  | Neighbors of proteasome endopeptidase complex |
|  | Neighbors of histone H3 |
|  | Neighbors of STAT5 |
|  | Neighbors of WNT |
|  | Neighbors of immunoglobulin |
|  | Neighbors of CD3 complex |
|  | Neighbors of TORC1 |
|  | Model of PPARG signaling in psoriasis |
|  | PPARG negative regulators and targets |
|  | Model of PPARG related pathways in psoriasis (short version) |
|  | IFN signaling in alveolar cells |
|  | Overview. Diseases of the digestive system (chapter 10) |
|  | 1\_Differentiation of psoriatic T cells |
|  | 4\_2\_Genes with mutations currently associated with ulcerative colitis |
|  | 4\_1\_Polymorphisms associated with inflammatory bowel diseases |
|  | Differentiation of psoriatic T cells |

---

|  |  |
| --- | --- |
| MedScan ID | 29939 |

---

|  |  |
| --- | --- |
| LocusLink ID | 29939 |
|  | 30009 |
|  | 57765 |
|  | 303496 |

---

|  |  |
| --- | --- |
| Alias | TBT I |
|  | OTTMUSP00000002150 |
|  | TBX21 |
|  | protein T bet |
|  | T-box expressed in T cells |
|  | transcription factor TBLYM |
|  | T-bet protein |
|  | T bet protein |
|  | T-cell-specific T-box transcription factor |
|  | T-bet |
|  | T-box transcription factor TBX21 |
|  | transcription factor T bet |
|  | T-PET |
|  | t-box transcription factor |
|  | TBET |
|  | T box expressed in T cells transcription factor |
|  | T-box 21 |
|  | T-cell-specific T-box transcription factor T-bet |
|  | T-box transcription factor 21 |
|  | RP23-428N4.9 |
|  | TBLYM |
|  | T-box protein 21 |
|  | TBT1 |
|  | Tbx21\_predicted |

---

|  |  |
| --- | --- |
| GO ID | 0001228 |
|  | 0003700 |
|  | 0000981 |
|  | 0001227 |
|  | 0001102 |
|  | 0000977 |
|  | 0043565 |
|  | 0044212 |
|  | 0030217 |
|  | 0001708 |
|  | 0071310 |
|  | 0072676 |
|  | 0007275 |
|  | 2000320 |
|  | 2000329 |
|  | 2000552 |
|  | 0032703 |
|  | 0000122 |
|  | 0045892 |
|  | 0048304 |
|  | 0045944 |
|  | 0045893 |
|  | 0043161 |
|  | 0045580 |
|  | 0006355 |
|  | 0009615 |
|  | 0043025 |
|  | 0005634 |
|  | 0003677 |
|  | 0010628 |
|  | 0050776 |
|  | 0006351 |

---

|  |  |
| --- | --- |
| KEGG ID | hsa:30009 |
|  | mmu:57765 |
|  | rno:303496 |

---

|  |  |
| --- | --- |
| Organism | Homo sapiens {Organism urn:agi-taxid:9606} |
|  | Mus musculus {Organism urn:agi-taxid:10090} |
|  | Rattus norvegicus {Organism urn:agi-taxid:10116} |
|  | Mus musculus |
|  | Homo sapiens |
|  | Rattus norvegicus |

---

|  |  |
| --- | --- |
| Mouse chromosome position | 11 |

---

|  |  |
| --- | --- |
| OMIM ID | 604895 |
|  | 208550 |

---

|  |  |
| --- | --- |
| Rat chromosome position | 10q31 |

---

|  |  |
| --- | --- |
| Hugo ID | 11599 |
|  | HGNC:11599 |

---

|  |  |
| --- | --- |
| Human chromosome position | 17q21.32 |

---

|  |  |
| --- | --- |
| Swiss-Prot Accession | Q9UL17 |
|  | Q9UL17.1 |
|  | Q9JKD8 |
|  | Q9JKD8.1 |
|  | D3ZCM2 |
|  | Q3U150 |
|  | Q9R0A6 |
|  | Q53EK5 |
|  | Q5PSB0 |

---

|  |  |
| --- | --- |
| GenBank ID | NC\_000017 |
|  | NM\_013351 |
|  | NP\_037483 |
|  | NG\_012166 |
|  | AC015674 |
|  | CH471109 |
|  | EAW94804 |
|  | GN337430 |
|  | CAY39299 |
|  | GN337449 |
|  | CAY39301 |
|  | AF093098 |
|  | AAF00055 |
|  | AF241243 |
|  | AAF61243 |
|  | AK223634 |
|  | BAD97354 |
|  | BC039739 |
|  | AAH39739 |
|  | Q9UL17 |
|  | NC\_000077 |
|  | NM\_019507 |
|  | NP\_062380 |
|  | AL606664 |
|  | AL627445 |
|  | BX255873 |
|  | CH466556 |
|  | EDL16071 |
|  | GN337432 |
|  | CAY39300 |
|  | GN337451 |
|  | CAY39302 |
|  | AF093099 |
|  | AAF00056 |
|  | AF241242 |
|  | AAF61242 |
|  | AK054495 |
|  | AK156271 |
|  | BAE33650 |
|  | AY836551 |
|  | AAV83787 |
|  | BC137986 |
|  | AAI37987 |
|  | BC137988 |
|  | AAI37989 |
|  | Q9JKD8 |
|  | NC\_005109 |
|  | NM\_001107043 |
|  | NP\_001100513 |
|  | AC\_000078 |
|  | AABR07030424 |
|  | AABR07030425 |
|  | AAHX01065830 |
|  | AAHX01065831 |
|  | AAHX01065832 |
|  | CH473948 |
|  | EDM05847 |
|  | ABBA01041142 |
|  | XP\_011523000 |
|  | XP\_011523001 |
|  | XM\_011524699 |
|  | XM\_011524698 |
|  | AAHY01095630 |
|  | NC\_018928 |
|  | CAM26989 |
|  | AMYH02033526 |
|  | AABR06065461 |
|  | CAM24010 |
|  | AABR06065460 |
|  | AC\_000149 |
|  | AC\_000033 |
|  | NT\_010783 |
|  | NW\_001838448 |
|  | NW\_926894 |
|  | AC\_000060 |
|  | Q53EK5 |
|  | NT\_165773 |
|  | NW\_001030424 |
|  | Q3U150 |
|  | Q5PSB0 |
|  | NW\_001084656 |
|  | NW\_047338 |

---

|  |  |
| --- | --- |
| Swiss-Prot ID | TBX21\_HUMAN |
|  | TBX21\_MOUSE |
|  | Q3U150\_MOUSE |
|  | D3ZCM2\_RAT |

---

|  |  |
| --- | --- |
| Cell Localization | Nucleus |

---

|  |  |
| --- | --- |
| Ensembl ID | ENSG00000073861 |
|  | ENSP00000177694.1 |
|  | ENST00000177694.2 |
|  | ENSMUSG00000001444 |
|  | ENSMUSP00000001484.2 |
|  | ENSMUST00000001484.2 |
|  | ENSRNOG00000009427 |
|  | ENSRNOP00000012538.5 |
|  | ENSRNOT00000012538.5 |
|  | ENST00000177694.1 |
|  | ENSMUSP00000001484 |
|  | ENSRNOT00000012538 |
|  | ENSRNOP00000012538 |
|  | ENSMUST00000001484 |
|  | ENSP00000177694 |
|  | ENST00000177694 |

---

|  |  |
| --- | --- |
| MGI ID | MGI:1888984 |
|  | 1888984 |

---

|  |  |
| --- | --- |
| RGD ID | 1308264 |

---

|  |  |
| --- | --- |
| Unigene ID | Hs.272409 |
|  | Mm.94519 |
|  | Rn.144930 |
|  | Mm.490611 |
|  | Mm.485627 |

---

|  |  |
| --- | --- |
| Homologene ID | 8353 |

---

|  |  |
| --- | --- |
| Shape | O-vertex |

---

|  |  |
| --- | --- |
| IPI ID | IPI00007732 |
|  | IPI00311393 |
|  | IPI00370968 |

---
